# Detection of Ergotized Rye

**Published:** 1857-04

**Authors:** 


### DETECTION OF ERGOTISED RYE.

To detect ergotised grains in corn, M. Payen gives the following instructions :—The ears affected are distinguishable by many of the grains in it being replaced by a violet-brown substance, almost black, of larger volume and frequently twisted, brittle, having a grey mass inside. The ergot may be distinguished even when no larger than the healthy grain, or when broken into several pieces, not only by its external dark colour, but also by its lightness; it floats on water, whereas the healthy grains sink to the bottom.

One-eighth to one-tenth per cent. of ergot in bread may cause gangrene and loss of the limbs, and the poisonous effect is more powerful on animals than on man. In poultry, the phalanges fall off; even the beak is detached. In pigs, the nails fall off, and the animal dies.

The dangers of ergot may be avoided by properly cleansing the grain, by the hand, by sifting, or by fanning. These various processes would be inexpensive, as the ergot would produce profit, sold for medicinal purposes. (*Journal de Chimie Médicale,* Dec. 1856, and *Chemist,* Jan. 1857.)

